# Prevalence, predictors, and management of advanced HIV disease among individuals initiating ART in Senegal, West Africa

**DOI:** 10.1186/s12879-019-3826-5

**Published:** 2019-03-15

**Authors:** Noelle A. Benzekri, Jacques F. Sambou, Sanou Ndong, Ibrahima Tito Tamba, Dominique Faye, Mouhamadou Baïla Diallo, Jean Phillippe Diatta, Khadim Faye, Ibrahima Sall, Fatima Sall, Noël Magloire Manga, Jean Jacques Malomar, Cheikh T. Ndour, Stephen E. Hawes, Moussa Seydi, Geoffrey S. Gottlieb

**Affiliations:** 1 Department of Medicine, University of Washington, Box 358061, 750 Republican St, Seattle, WA 98109-4725 USA; 2 Centre de Santé de Ziguinchor, Ziguinchor, Senegal; 3 Service des Maladies Infectieuses et Tropicales, Centre Hospitalier National Universitaire (CHNU) de Fann, Dakar, Senegal; 4 Centre de Santé de Bignona, Bignona, Senegal; 5 Hôpital de la Paix, Ziguinchor, Senegal; 6 Division de Lutte contre le Sida et les IST, Ministère de la Santé et de l’Action Sociale, Dakar, Sénégal; 7 Department of Epidemiology, University of Washington, Seattle, WA USA; 8 Department of Global Health, University of Washington, Seattle, WA USA

**Keywords:** HIV/AIDS, Antiretroviral therapy (ART), Advanced disease, World Health Organization (WHO), Prophylaxis, Preventive therapy, Implementation, Co-trimoxazole, Isoniazid preventive therapy (IPT), Tuberculosis (TB), Fluconazole, Cryptococcal meningitis, Traditional healer, Senegal, West Africa

## Abstract

**Background:**

The WHO guidelines for the management of advanced HIV disease recommend a package of care consisting of rapid initiation of antiretroviral therapy (ART), enhanced screening and diagnosis of tuberculosis (TB) and cryptococcal meningitis, co-trimoxazole prophylaxis, isoniazid preventive therapy (IPT), fluconazole pre-emptive therapy, and adherence support. The goals of this study were to determine the prevalence of advanced HIV disease among individuals initiating ART in Senegal, to identify predictors of advanced disease, and to evaluate adherence to the WHO guidelines.

**Methods:**

This study was conducted among HIV-positive individuals initiating ART in Dakar and Ziguinchor, Senegal. Clinical evaluations, laboratory analyses, questionnaires and chart review were conducted. Logistic regression was used to identify predictors of advanced disease.

**Results:**

A total of 198 subjects were enrolled; 70% were female. The majority of subjects (71%) had advanced HIV disease, defined by the WHO as a CD4 count < 200 cells/mm^3^ or clinical stage 3 or 4. The median CD4 count was 185 cells/mm^3^. The strongest predictors of advanced disease were age ≥ 35 (OR 5.80, 95%CI 2.35–14.30) and having sought care from a traditional healer (OR 3.86, 95%CI 1.17–12.78). Approximately one third of subjects initiated ART within 7 days of diagnosis. Co-trimoxazole prophylaxis was provided to 65% of subjects with CD4 counts ≤350 cells/mm^3^ or stage 3 or 4 disease. TB symptom screening was available for 166 subjects; 54% reported TB symptoms. Among those with TB symptoms, 39% underwent diagnostic evaluation. Among those eligible for IPT, one subject received isoniazid. No subjects underwent CrAg screening or received fluconazole to prevent cryptococcal meningitis.

**Conclusions:**

This is the first study to report an association between seeking care from a traditional healer and presentation with WHO defined advanced disease in sub-Saharan Africa. Given the widespread use of traditional healers in sub-Saharan Africa, future studies to further explore this finding are indicated. Although the majority of individuals in this study presented with advanced disease and warranted management according to WHO guidelines, there were numerous missed opportunities to prevent HIV-associated morbidity and mortality. Programmatic evaluation is needed to identify barriers to implementation of the WHO guidelines and enhanced funding for operational research is indicated.

## Background

Progress towards reducing AIDS-related deaths in Western and Central Africa lags behind other regions. Nearly one third of global HIV-related deaths occur in Western and Central Africa, and although globally HIV-related deaths have declined by 34%, deaths in Western and Central Africa have declined by only 24%, from 370,000 in 2010 to 280,000 in 2017 [1]. According to UNAIDS estimates, only 48% of people living with HIV (PLHIV) in Western and Central Africa know their status, indicating a substantial regional gap in achieving the first of the 90–90-90 targets to end the AIDS epidemic [2]. Delayed diagnosis contributes to ongoing presentation with advanced HIV disease and HIV-associated mortality.

Despite efforts to improve access to HIV testing and treatment, approximately one third of individuals with HIV in sub-Saharan Africa (SSA) present with advanced disease [3–5]. In West Africa specifically, more than half of PLHIV present with advanced disease. The median CD4 count at ART initiation is 186 cells/mm^3^, which is lower than other regions on the continent and is indicative of severe immunodeficiency and susceptibility to opportunistic infections [3].

In 2017, the WHO published guidelines recommending a package of targeted interventions to reduce morbidity and mortality among people presenting with advanced HIV [6]. The guidelines describe a package of care which includes rapid initiation of ART, enhanced screening and diagnosis of tuberculosis (TB) and cryptococcal meningitis (CM), co-trimoxazole prophylaxis, isoniazid preventive therapy (IPT), and fluconazole pre-emptive therapy. The goals of this study were to determine the prevalence of advanced disease among individuals initiating ART in Senegal, West Africa, to identify predictors of advanced disease, and to evaluate adherence to the WHO guidelines for the management of advanced HIV disease.

## Methods

This study was conducted at HIV testing and treatment sites located at the Centre Hospitalier National Universitaire de Fann (Centre Régional de Recherche et de Formation à la Prise en Charge Clinique de Fann) in Dakar and the Centre de Santé de Ziguinchor, located in Ziguinchor. Dakar is the urban capital of the country, while Ziguinchor is located in the southern Casamance region. All HIV-positive individuals initiating ART through the Senegalese National AIDS program (ISAARV) who provided written informed consent were eligible for enrollment. For subjects < 18 years of age, consent was obtained from their legal guardian. Subjects were enrolled sequentially from April 2017 to April 2018. Study procedures were approved by the University of Washington Institutional Review Board and the Senegal Comité National d’Ethique pour la Recherche en Santé (CNERS).

Study encounters were conducted in the participant’s preferred language, including French, Wolof, Diola, Peular, Mandinka, or Creole. Sociodemographic characteristics were determined using interviewer administered questionnaires. Food insecurity was determined using the Household Food Insecurity Access Scale [7]. WHO clinical staging was determined by clinical evaluation and chart review [8]. A review of systems was performed during clinical evaluation. Laboratory testing was conducted to determine HIV type and to measure CD4 cell count. Days until ART initiation was calculated by subtracting the date of ART initiation from the date of HIV diagnostic testing. Medical records were reviewed to capture clinical presentation, medical history, diagnostic testing, and medications prescribed.

Data were analyzed using SPSS Statistics 23 (IBM, Armonk, N.Y.). Descriptive analysis was performed for all variables. Logistic regression was used to identify predictors of presentation with advanced disease. Variables identified in the literature as associated with advanced disease were evaluated using simple regression, Variables which were predictive using simple regression were included in the multiple regression model. Missing data were excluded from analysis. *P*-values < 0.05 were considered significant.

## Results

A total of 198 subjects were enrolled, 83 (42%) in Dakar and 115 (58%) in Ziguinchor (Table 1). This represents 65% of the 303 individuals that initiated ART at the study sites during the study period. The majority of subjects (89%) were infected with HIV-1, including two HIV-1/HIV-2 dually infected subjects, and 21 (11%) were infected solely with HIV-2. More than two thirds of subjects (70%) were female. Approximately 58% of subjects were ≥ 35 years of age. Nearly two thirds reported that they live in either the County of Dakar or the County of Ziguinchor. Nearly one third (31%) had no formal education and only 29% had ever attended secondary school. The median number of children per subject was 2, with a range of 0–10. Most subjects were married, 39% were in monogamous marriages and 16% were in polygamous marriages. Only 14% of subjects were employed and the majority (69%) were food insecure. One third reported seeking care from a traditional healer prior to presentation.

**Table 1:** Subject characteristics of HIV positive individuals in Dakar and Ziguinchor presenting for HIV care (*N* = 198)

	N (%)
Number of subjects	198
Clinic site	
Dakar	83 (41.9)
Ziguinchor	115 (58.1)
HIV type	
HIV-1 or dual^a^	165 (88.7)
HIV-2	21 (11.3)
Female	124 (70.1)
Age categories	
< 18	6 (3.5)
18–34	67 (39.0)
≥ 35	99 (57.6)
Residence in County of Dakar or Ziguinchor	117 (63.6)
Highest level of formal education	
none	47 (30.7)
primary	62 (40.5)
secondary	36 (23.5)
university	8 (5.2)
Number of children, median (range; IQR)	2 (0–10; 1–4)
Marital status	
single	24 (14.0)
monogamous	66 (38.6)
polygamous	27 (15.8)
divorced	28 (16.4)
widowed	26 (15.2)
Employed	23 (14.1)
Food insecure	113 (68.5)
Ever sought care from a Traditional Healer	53 (33.8)

*IQR* interquartile range

^a^Includes 2 HIV-1/HIV-2 dually infected subjects

CD4 cell counts were available for 185 subjects (Table 2). The median CD4 count at presentation was 185 cells/mm^3^, with a range of 1–1541. The median CD4 cell count differed among those who were infected with HIV-1 versus those were infected solely with HIV-2 (170 cells/mm^3^ vs. 412 cells/mm^3^, *p* = 0.03). Nearly three quarters of subjects presented with a CD4 count ≤350 cells/mm^3^, 55% presented with < 200 cells/mm^3^, 36% had < 100 cells/mm^3,^ and 20% had < 50 cells/mm^3^. WHO clinical staging was available for 167 subjects, of which 53% had WHO stage 3 or 4 disease. The most common WHO stage 3 conditions were severe weight loss, chronic diarrhea, oral candidiasis, and pulmonary TB. The most common WHO stage 4 condition was extra-pulmonary TB. The majority of subjects (71%) had advanced HIV disease, defined by the WHO as a CD4 count < 200 cells/mm^3^ or stage 3 or 4 disease.

**Table 2:** Prevalence of advanced HIV disease

	N (%)
All subjects^a^, median CD4 cell count (range)	185 (1–1541)
HIV-1 infected^b^, median CD4 cell count (range)	170 (1–1541)*
HIV-2 infected, median CD4 cell count (range)	412 (9–1005)*
CD4 cell count categories	
≤ 350 cells/mm^3^	135 (73.0)
< 200 cells/mm^3^	102 (55.1)
< 100 cells/mm^3^	67 (36.2)
< 50 cells/mm^3^	36 (19.5)
WHO stage 3 or 4^c^	89 (53.3)
Stage 3 conditions	
Severe weight loss	35 (21.2)
Chronic diarrhea	29 (17.6)
Oral candidiasis	28 (17.0)
Oral hairy leukoplakia	5 (3.0)
Pulmonary TB	19 (13.1)
Severe bacterial infections	5 (3.0)
Stage 4 conditions	
Wasting	6 (3.6)
PCP	2 (1.2)
Recurrent severe bacterial PNA	2 (1.2)
Esophageal candidiasis	6 (3.6)
Extrapulmonary TB	12 (7.3)
Kaposi sarcoma (cutaneous)	3 (1.8)
Toxoplasmosis	1 (0.6)
Extrapulmonary cryptococcosis	2 (1.2)
Invasive cervical carcinoma	1 (0.6)
CD4 count < 200 cells/mm^3^ or WHO stage 3 or 4^d^	123 (71.1)

^*^The difference in CD4 cell counts was statistically significant, *p*-value = 0.03

^a^CD4 cell counts available for 185 subjects

^b^Includes 2 dually infected subjects

^c^WHO stage available for 167 subjects; Specific WHO stage 3 or 4 conditions available for 165 subjects

^d^Data available for 173 subjects

Variables which were predictive of advanced disease using simple regression included male sex, age ≥ 35, and having sought care from a traditional healer prior to presentation (Table 3). Clinic site, residence in Dakar or Ziguinchor, education, number of children, marital status, employment status, and food insecurity were not associated with advanced disease. In the multiple regression model, age ≥ 35 (OR 5.80, 95% CI 2.35–14.30) and having sought care from a traditional healer prior to presentation (OR 3.86, 95% CI 1.17–12.78) were predictive of advanced disease.

**Table 3:** Predictors of advanced HIV disease (CD4 count < 200 cells/mm^3^ OR WHO stage 3 or 4)^a^

Simple regressions					Multiple regression			
	OR	95% CI		*p*-value	OR	95% CI		*p*-value
Male	**2.45**	**1.02**	**5.87**	**0.04**	2.13	0.76	5.96	0.15
Age ≥ 35 (ref. <35)	**7.67**	**3.26**	**18.02**	**< 0.01**	**5.80**	**2.35**	**14.30**	**< 0.01**
Clinic site (ref. Ziguinchor)	1.33	0.64	2.77	0.45	–	–	–	–
Residence (ref. Dept. of Dakar or Ziguinchor)	0.77	0.36	1.66	0.51	–	–	–	–
Education (ref. none)								
primary	0.68	0.22	2.17	0.52	–	–	–	–
secondary or university	0.39	0.12	1.26	0.12	–	–	–	–
Number of children	1.07	0.90	1.29	0.44	–	–	–	–
Marital status (ref. monogamous)								
single	1.06	0.37	3.00	0.91	–	–	–	–
polygamous	0.70	0.22	2.23	0.54	–	–	–	–
divorced	1.67	0.54	5.17	0.38	–	–	–	–
widowed	2.16	0.56	8.43	0.27	–	–	–	–
Employed	1.33	0.45	3.94	0.61	–	–	–	–
Food insecure	1.54	0.70	3.39	0.29	–	–	–	–
Sought care from a traditional healer	**5.04**	**1.64**	**15.51**	**0.01**	**3.86**	**1.17**	**12.78**	**0.03**

^a^Among HIV-1 infected subjects ≥18 years old

The most frequently prescribed antiretrovirals were tenofovir (TDF) (95%), lamivudine or emtricitabine (3TC or FTC) (100%), and efavirenz (EFV) (84%) (Table 4). One HIV-2 infected subject received efavirenz. Twenty-one subjects received lopinavir/ritonavir including 18 HIV-2 infected subjects, 1 dually infected subject receiving TB treatment during an abacavir (ABC) stock-out, and 2 non-pregnant adults with HIV-1 infection. Integrase inhibitors were not available. Among those with no known active TB or CM, 34% initiated ART within 7 days of diagnosis. Among those with advanced disease, 38% initiated ART within 7 days of diagnosis. Co-trimoxazole prescription data were available for 157 subjects. Co-trimoxazole prophylaxis was provided to 53% of all subjects and 65% of subjects with CD4 counts ≤350 cells/mm^3^ or stage 3 or 4 disease. In one case, a co-trimoxazole stock-out was reported. A review of symptoms was available for 166 subjects, 77 (46%) did not report symptoms consistent with TB (fever, cough, weight loss, night sweats) and 89 (54%) reported symptoms consistent with TB. Among those without TB symptoms, 4 had extra-pulmonary TB, resulting in 73 (44%) of subjects eligible for IPT. Among those eligible for IPT, one subject (1%) received a 2 week prescription for INH. Among those with TB symptoms, only 35 (39%) underwent diagnostic evaluation, 31 were evaluated by microscopy for acid-fast bacilli using Ziehl-Neelsen staining and 10 were evaluated by Xpert® MTB/RIF (Cepheid). There was no rifampicin resistance detected. The diagnostic evaluation was negative for 25 (71%) of the subjects who were evaluated. Among non-pregnant subjects ≥18 years of age, a review of symptoms and CD4 counts were available for 133. There were 46 subjects (35%) with CD4 cell counts < 100 and no neurological symptoms reported. No subjects underwent cryptococcal antigen (CrAg) screening and none received fluconazole to prevent CM.

**Table 4:** Management of advanced HIV disease

	N (%)
ART	
Components of ART regimen^a^	
NRTI backbone	
TDF + 3TC or FTC	160 (95.2)
AZT + 3TC or FTC	8 (4.8)
EFV-based regimen^b^	141 (83.9)
NVP-based regimen	3 (1.8)
Lop/rit-based regimen^c^	21 (12.5)
Triple NRTI regimen using ABC	3 (1.8)
Number of days between diagnosis and initiation of ART, median (IQR)	13.0 (6.0-26.5)
ART started within ≤7 days of diagnosis^d^	
Among all subjects	42 (33.9)
Among those with advanced disease	29 (37.7)
Co-trimoxazole	
Among all subjects^e^	83 (52.9)
Among those with CD4 ≤350 cells/mm3 OR stage 3 or 4 disease^f^	77 (64.7)
IPT	
No TB symptoms present^g^	77 (46.4)
Active extra-pulmonary TB	4 (5.2)
Eligible for IPT: No TB screening symptoms or active TB	73 (44.0)
INH prescription provided^h^	1 (1.4)
TB symptoms present	89 (53.6)
Diagnostic evaluation performed	35 (39.3)
Diagnostic evaluation negative^i^	25 (71.4)
Fluconazole	
CD4 <100 and neurological symptoms present	7 (5.3)
CD4 <100 and no neurological symptoms present^j^	46 (34.6)
CrAg screening	0 (0)
Fluconazole prescription provided	0 (0)

^a^Regimen available for 168 subjects

^b^Includes 1 HIV-2 infected subject

^c^Includes 18 HIV-2 infected subjects, 1 dually infected subject receiving TB treatment during an ABC stock-out, and 2 HIV-1 infected subjects

^d^Excluding those with active TB or CM

^e^Data available for 157 subjects

^f^Data available for 151 subjects, of which 119 met criteria

^g^Data available for 166 subjects

^h^INH provided for 2 weeks

^i^Eligibility for IPT in this scenario is dependent on clinical presentation and likelihood of TB

^j^Among non-pregnant subjects ≥18 years of age. Data available for 133 subjects

## Discussion

In this study, conducted in Senegal, West Africa, the majority of individuals presented with advanced HIV disease, defined as a CD4 count < 200 cells/mm^3^ or clinical stage 3 or 4, and therefore warranted care according to the WHO guidelines for the management of advanced HIV. The WHO guidelines recommend a package of care consisting of rapid initiation of ART, enhanced screening and diagnosis of TB and CM, co-trimoxazole prophylaxis, IPT, fluconazole pre-emptive therapy, and adherence support [6, 9].

Provision of a package of care for people with advanced HIV disease is supported by data from two randomized trials of intervention packages for individuals with advanced disease in SSA [10, 11]. The first study, conducted among individuals with a CD4 cell count < 200 initiating ART in Tanzania and Zambia, evaluated a package consisting of CrAg screening with fluconazole pre-emptive therapy and adherence support compared to the standard of care. The intervention package resulted in a reduction in mortality from 18% in the standard of care group to 13% in the intervention group. The second study, conducted among individuals starting ART in Kenya, Uganda, Malawi, and Zimbabwe with CD4 counts < 100, evaluated an enhanced package consisting of co-trimoxazole, IPT, azithromycin, albendazole, and primary fluconazole prophylaxis without CrAg screening, compared to the standard of care (co-trimoxazole alone). Enhanced prophylaxis resulted in reduced death rates at 24 weeks compared to the standard of care (8.9% versus 12.2%; *p* = 0.03).

According to the WHO guidelines, “rapid initiation of ART should be offered to all PLHIV following a confirmed diagnosis and clinical assessment” with the exception of individuals suspected of having TB or CM [6]. Rapid initiation is defined as within 7 days from the day of HIV diagnosis, and should be prioritized among those with advanced disease. In our study, rapid initiation of ART occurred in only 34% of eligible subjects and 38% of those with advanced disease. When ART was initiated, the majority received WHO recommended first-line agents [9]. Contraindicated or sub-optimal regimens were given to 4 subjects, including the provision of efavirenz to a subject infected with HIV-2, treatment with lopinavir/ritonavir in an HIV-2 infected subject receiving treatment for TB, and treatment with lopinavir/ritonavir to two non-pregnant adults infected with HIV-1. Approximately 20% of HIV-infections in Senegal are due to HIV-2, which is intrinsically resistant to NNRTIs, including efavirenz. Protease inhibitors are part of first line treatment for HIV-2 and dually infected subjects in Senegal; individuals who are infected with HIV-2 and receiving treatment for TB receive triple NRTI therapy. Although integrase inhibitors are included as first line options in the 2016 WHO guidelines, they are currently not accessible in most settings in Senegal.

Rapid initiation of ART has the potential to improve ART uptake, retention in care and viral suppression, and to decrease disease progression, HIV transmission, and mortality [6, 12–14]. Barriers to early initiation of ART that have been reported in other regions of SSA include systems-based factors, such as requirements for pre-ART counseling sessions and multiple visits for laboratory testing, insufficient healthcare worker knowledge about HIV care, and ART stock-outs [6, 13, 15–17]. Among pregnant women in Kenya and Malawi, patient reported barriers include the need to seek approval from their husbands, and insufficient time to disclose their status with consequent potential for stigma, conflict, and domestic violence [18, 19]. Barriers to rapid initiation of ART in Senegal have not been reported, and it is possible that these factors differ depending on the setting. Site specific evaluations to identify systems-based and patient-based barriers and facilitators to rapid initiation of first line ART are warranted.

Notably, we did not find an association between advanced disease and clinic site, residence, education, number of children, marital status, employment, or food insecurity. The strongest predictors of advanced disease were age ≥ 35 years and having sought care from a traditional healer. To our knowledge, this is the first study to report an association between seeking care from a traditional healer and presentation with WHO defined advanced disease in SSA. Given the widespread use of traditional healers in SSA [20, 21], this finding may have important implications for HIV programs. Future studies to further explore this finding are indicated.

The WHO criteria for co-trimoxazole prophylaxis among PLHIV are CD4 count ≤350 cells/mm^3^ or clinical stage 3 or 4, or lifelong co-trimoxazole for any CD4 count in settings with a high prevalence of malaria or bacterial infections. An area of high malaria transmission is defined as > 1 case/1000 population per year [22]. Malaria is endemic in Senegal and 99% of the population is considered at high risk of infection [23]. The risk is especially high in the Casamance region where there were up to 200 confirmed cases per 1000 population in 2016. Based on these indicators, all PLHIV in Senegal should receive lifelong co-trimoxazole. Co-trimoxazole prophylaxis has been shown to reduce mortality in PLHIV by up to 45% and is well-established as an integral component of HIV care [24–28]. Nearly half of the individuals in this study did not receive co-trimoxazole prophylaxis, including one third of those with advanced disease. There is a critical need to identify barriers to the implementation of this intervention, which costs just a few cents day per day and is known to substantially decrease morbidity and mortality among PLHIV [24].

Tuberculosis remains the leading cause of death among PLHIV, responsible for one third of deaths among PLHIV globally [29, 30]. Strategies to reduce TB-associated mortality among PLHIV include increased TB case finding, IPT, and infection control. According to WHO guidelines, routine TB symptom screening using an algorithm containing fever, cough, weight loss and night sweats, should be used to help identify HIV-infected individuals who should undergo expedited diagnostic evaluation for TB, and those who should receive IPT. Furthermore, diagnostic evaluation with sputum Xpert MTB/RIF, rather than traditional microscopy, should be used as the initial test among symptomatic PLHIV. The lateral flow urine lipoarabinomannan assay (LF-LAM) may be used among symptomatic individuals with a CD4 count ≤100 or those who are severely ill [6, 9]. Among those who do not have any of the screening symptoms, active TB is considered unlikely and IPT should be offered.

Approximately half of the subjects in this study reported symptoms consistent with TB. Among those who were symptomatic, only 39% underwent diagnostic evaluation and for the majority, traditional microscopy was utilized. These findings indicate a need to identify barriers to diagnostic evaluation and to improve access to WHO recommended diagnostic tests. Importantly, among those who did not have TB symptoms, only one individual was provided with isoniazid (INH). That individual received a 2 week prescription for INH, although the WHO recommends a minimum of 6 months of IPT. The provision of IPT is recommended by the Senegal National AIDS program. Although the reasons for poor IPT uptake in Senegal have not been evaluated, barriers may include provider fear of undiagnosed TB, concerns about drug interactions, adverse effects, or medication adherence, uncertainty about eligibility criteria, or lack of awareness of national and international policies. A survey of physicians and healthcare workers involved in the care of PLHIV in Senegal could serve to elucidate these factors.

Cryptococcal meningitis (CM) is a leading cause of death among individuals with advanced HIV, accounting for 15–20% of global AIDS-related deaths [6, 31]. CrAg screening combined with pre-emptive anti-fungal therapy (PET) with fluconazole has the potential to reduce CM associated mortality [10, 32–36]. The WHO recommended package of care for individuals with advanced disease includes CrAg screening for individuals with a CD4 count < 100 followed by fluconazole for CrAg positive people without evidence of meningitis. The 2018 WHO guidelines for the diagnosis, prevention and management of cryptococcal disease recommend that CrAg screening be performed for all HIV-positive adults with a CD4 count < 100 cells/mm^3^ and that screening should be considered at a CD4 count threshold of < 200 cells/mm^3^ [31]. When CrAg screening is not available, the recent guidelines state that fluconazole primary prophylaxis should be given to adults and adolescents living with HIV who have a CD4 cell count < 100 cells/mm^3^ (strong recommendation; moderate-certainty evidence) and may be considered at a higher CD4 cell count threshold of < 200 cells/mm^3^ (conditional recommendation; moderate-certainty evidence). Neither CrAg screening combined with PET nor fluconazole prophylaxis were provided to any of the subjects evaluated in our study. CrAg screening is not routinely available in Senegal and the recommendation for prophylaxis in the absence of screening was only recently released. If implemented, these strategies could provide important opportunities to decrease morbidity and mortality due to CM among PLHIV in Senegal.

The primary limitation of our study is sample size. Turnover of healthcare personnel and regional strikes involving healthcare personnel limited our ability to enroll all individuals who initiated ART during the study period. This was not a population-based study and may not be representative of PLHIV or those presenting for care overall in Dakar or Ziguinchor or more broadly in Senegal. Our study is further limited by incomplete data; completion of standardized national forms for the evaluation and management of HIV was inconsistent. A strength of our study is that we sequentially enrolled HIV-positive individuals initiating ART at two study sites in Senegal to capture a sample that is representative of the HIV-positive population and provide an evaluation of the management of advanced HIV disease that represents national practices.

Senegal was an early leader in responding to the HIV epidemic through the implementation of the first government ART treatment program in Africa in 1998. In the current era, proactive efforts to ensure adherence to WHO and Senegalese guidelines and access to evidence-based interventions for individuals with advanced HIV disease are similarly warranted.

## Conclusion

We evaluated the implementation of the WHO guidelines for the management of advanced HIV disease among individuals initiating ART in Senegal, West Africa. We found that although the majority of individuals presented with advanced disease and warranted management according to WHO guidelines, there were numerous missed opportunities to prevent HIV-associated morbidity and mortality. Programmatic evaluation is needed to identify barriers to implementation and enhanced funding for operational research is indicated.

## Abbreviations

3TC: Lamivudine
ABC: Abacavir
ART: Antiretroviral therapy
CM: Cryptococcal meningitis
CNERS: Comité National d’Ethique pour la Recherche en Santé
CrAg: Cryptococcal antigen
EFV: Efavirenz
FTC: Emtricitabine
HIV: Human Immunodeficiency Virus
INH: Isoniazid
IPT: Isoniazid Preventive Therapy
ISAARV: Initiative Sénégalaise d’Accès aux ARV
NNRTI: Non-nucleoside reverse transcriptase inhibitor
NRTI: Nucleoside reverse transcriptase inhibitor
PET: Pre-emptive anti-fungal therapy
PLHIV: People Living with HIV
SSA: Sub-Saharan Africa
TB: Tuberculosis
TDF: Tenofovir
WHO: World Health Organization
